# Multiple object individuation and subitizing in enumeration: a view from electrophysiology

**DOI:** 10.3389/fnhum.2015.00162

**Published:** 2015-04-02

**Authors:** Veronica Mazza, Alfonso Caramazza

**Affiliations:** ^1^Center for Mind/Brain Sciences (CIMeC), University of TrentoRovereto, Italy; ^2^IRCSS San Giovanni di Dio FatebenefratelliBrescia, Italy; ^3^Department of Psychology, Harvard UniversityCambridge, MA, USA

**Keywords:** object individuation, enumeration, EEG, subitizing, N2pc

## Abstract

What are the processes involved in determining that there are exactly *n* objects in the visual field? The core level of representation for this process is based on a mechanism that iteratively individuates each of the set of relevant objects for exact enumeration. In support of this proposal, we review recent electrophysiological findings on enumeration-at-a-glance and consider three temporally distinct responses of the EEG signal that are modulated by object numerosity, and which have been associated respectively with perceptual modulation, attention selection, and working memory. We argue that the neural response associated with attention selection shows the hallmarks of an object individuation mechanism, including the property of simultaneous individuation of a limited number of objects thought to underlie the behavioral subitizing effect. The findings support the view that the core component of exact enumeration is an attention-based individuation mechanism that binds specific features to locations and provides a stable representation of a limited set of relevant objects. The resulting representation is made available for further cognitive operations for exact enumeration.

## Object Individuation and Exact Enumeration

Theories of number processing (Mandler and Shebo, 1982; Feigenson et al., 2004; Piazza, 2010) have drawn a distinction between two different types of numerosity computation of briefly presented objects: estimation and exact enumeration. Estimation leads to an approximate representation of the number of objects in the visual field. This system is thought to compute an analog representation, as occurs for other sensory stimulus dimensions (e.g., Dehaene, 1997; Walsh, 2003; Burr and Ross, 2008). This latter aspect suggests that approximate enumeration relies on early perceptual mechanisms, which provide an imprecise and coarse representation of “spatially” separable entities. In contrast, exact enumeration requires a high level of precision based on a procedure that ensures that the cognitive system consider each of the elements to be enumerated once and only once (Trick and Pylyshyn, 1994). In other words, items need to be represented individually in order to be counted correctly. As for any other cognitive phenomenon, exact enumeration involves different levels of representation, starting from sensory processing up to the mapping of the computed numerosity onto a symbolic representation (Santens et al., 2010). In contrast to early models of enumeration and individuation (e.g., Trick and Pylyshyn, 1994), which assumed that individuation would take place at a pre-attentive level, here we argue that the core level of representation for this process is grounded on an attention-based mechanism that individuates relevant objects for exact enumeration.

**Figure 1:**
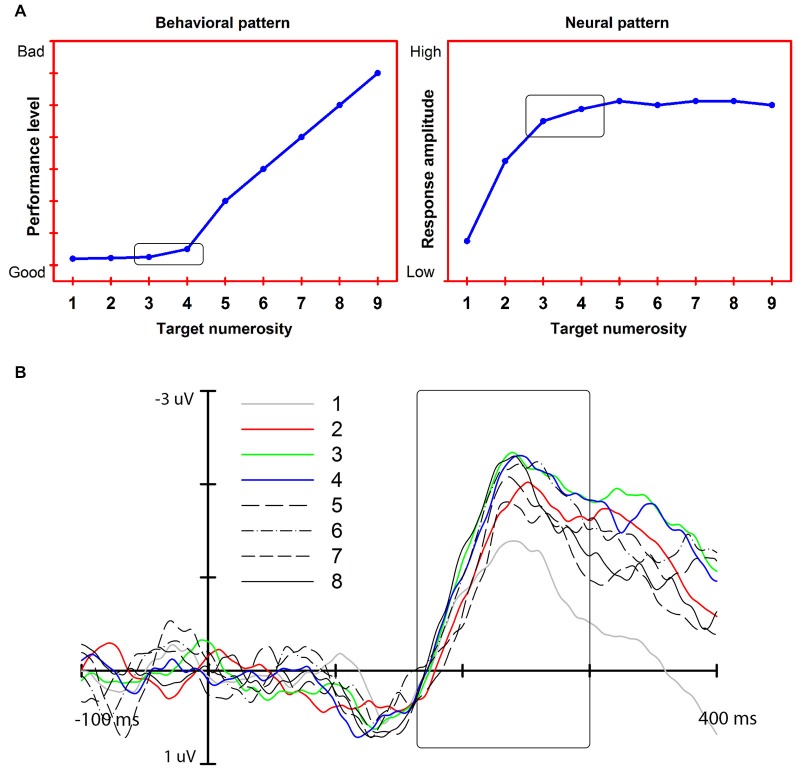
**(A)** Left: The subitizing effect (“typical” pattern, simulated data). Right: Predicted neural response associated with exact enumeration and the subitizing effect. **(B)** The N2pc response and its modulation as a function of target numerosity (adapted from Pagano et al., 2014). The modulation reaches an asymptote at approximately 3 target elements: the N2pc amplitude increases from 1 to 3–4 targets (colored lines). No further increase is measured for larger target numerosities (black lines).

Our account exploits a feature of exact enumeration: the subitizing effect. Subitizing is the fast and accurate enumeration of a small set of elements as compared to a slower and less accurate rate of enumeration time for larger sets of elements (Mandler and Shebo, 1982; Figure 1A). This effect is taken to reflect the functioning of a capacity-limited individuation mechanism that enables a precise representation of a maximum of 3–4 elements. To be efficient in all vision contexts, this mechanism should operate not only when the elements to be enumerated are presented in isolation but also when interspersed with non-relevant, distracting objects, as might occur in cluttered scenes (see Trick and Pylyshyn, 1993). We argue that recent electrophysiological (EEG) findings on enumeration-at-a-glance (Libertus et al., 2007; Hyde and Spelke, 2009, 2012; Mazza and Caramazza, 2011; Ester et al., 2012; Gebuis and Reynvoet, 2012; Pagano and Mazza, 2012; Anderson et al., 2013b; Mazza et al., 2013; Pagano et al., 2014) show that a component of the EEG signal that we have associated with single and multiple object individuation (see Mazza et al., 2009, 2013) is the mechanism that underlies the subitizing effect.

## Recent EEG Advances on Exact Enumeration

EEG has been used for decades to provide a time-wise evaluation of the functioning of the processing stages of various cognitive operations, from stimulus recognition to response execution, but only recently has it been used specifically to understand the functional mechanisms underlying exact enumeration. We argue that three effects should characterize the EEG responses that reflect the object individuation mechanism at the core of the exact enumeration process. The three effects are as follows: (1) since the object individuation mechanism is assumed to process multiple objects simultaneously (e.g., Pylyshyn, 2001) the neural response should be modulated by the number of objects individuated simultaneously; (2) as documented by seminal behavioral studies on subitizing (Kaufman et al., 1949; Mandler and Shebo, 1982) object individuation is limited to 3–4 co-occurring elements; therefore, the EEG response should reach asymptote at 3–4 elements, corresponding to the subitizing limit; and (3) behavioral studies (Trick and Pylyshyn, 1993; Watson et al., 2007) have found that object individuation occurs in both cluttered and uncluttered scenes; therefore, the previous two EEG effects should be found both when targets are presented in isolation and when they are presented together with irrelevant elements or distracters.

In this article, we consider temporally distinct EEG responses that are modulated by object numerosity and, thus, could be related to the subitizing phenomenon: an early response associated with perceptual modulation (N1), a middle, attention-related response that we associate with object individuation (N2pc), and a late response associated with visual working memory (CDA). Our review of the extant data indicates that the middle, attention-related response meets the three neural effects for the subitizing phenomenon, indicating that it is the earliest stage at which a visual representation appropriate for exact enumeration becomes available (Figure 1B).

### Early Neural Modulation of Object Numerosity

Recent studies (e.g., Nan et al., 2006; Libertus et al., 2007; Hyde and Spelke, 2009; Gebuis and Reynvoet, 2012) have shown that object numerosity modulates an early, posterior component of neural activity (N1, elicited at around 130 ms), which is typically ascribed to spatial attention. In particular, N1 modulations have been associated with enhanced processing of a stimulus at a pre-cued location in space (Mangun et al., 1993). In a series of passive viewing tasks, Hyde et al. (Hyde and Spelke, 2009, 2012; Hyde and Wood, 2011) showed that the N1 amplitude increases with object numerosity and reaches a plateau at approximately 3 elements (but see Nan et al., 2006; Libertus et al., 2007; for a higher numerical plateau). They interpreted this effect as reflecting the functioning of an object-tracking mechanism that individuates and computes discrete representations up to a maximum of 3 objects simultaneously (see Hyde, 2011).

**Figure 2:**
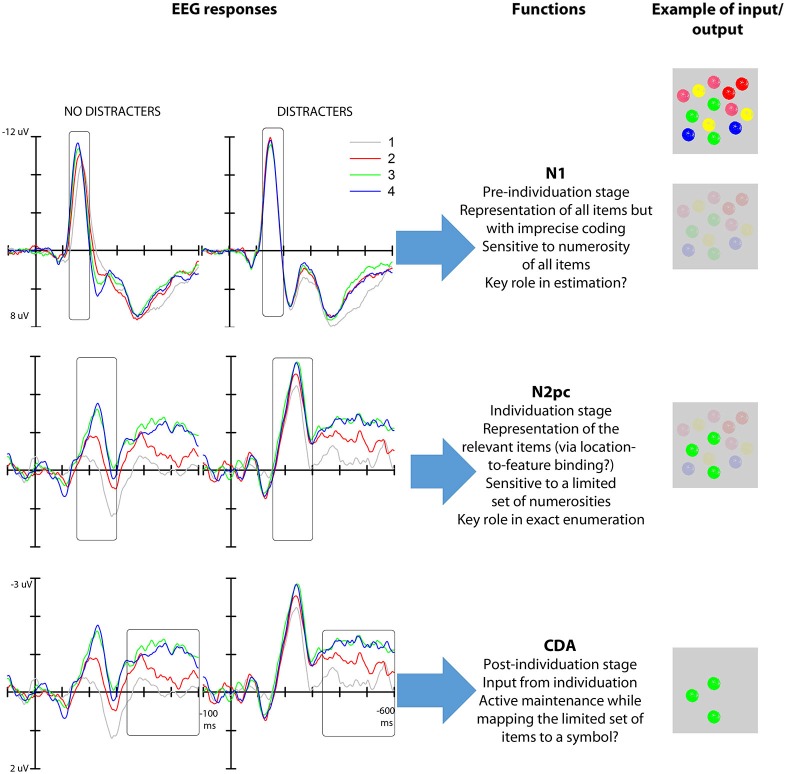
**Left: N1, N2pc and CDA modulations as a function of target numerosity both with and without distracters (data from Mazza et al., 2013)**. Middle: functional interpretation of the mechanisms reflected in the N1 (top), N2pc (middle) and CDA (bottom). Right: hypothetical output representation (given the input represented—multiple colored objects—in the first row) of the various stages of processing reflected in the three EEG responses. The first stage depicts a global representation were only location is represented distinctly; the second stage represents the clearly individuated target elements (green) among background elements; the third stage represents the selected elements in working memory.

Given the interpretation of the numerosity-based modulation of the N1 amplitude, if this neural response captured the individuation mechanism necessary for exact enumeration then it should be selectively engaged by the elements that are relevant for enumeration. On this view, the neural modulation of N1 is expected to track the portion of the total elements in a visual field that need to be individuated for the enumeration task. However, we have recently found (Mazza et al., 2013) that the modulation of the N1 is not restricted to target elements but occurs for all the objects in the visual field irrespective of whether they are part of the enumeration set; that is, the N1 modulation is not driven by the number of targets but by the total number of objects in a visual field, both targets and distracters, thereby failing to meet one of the criteria for an object individuation system (Figure 2). For this reason, we suggest that the representation produced at this stage of processing reflects the overall number of elements in the visual map—a representation that is not appropriate for the multiple-object individuation necessary for exact enumeration. We additionally speculate that this representation may be sufficient for estimation, although future work will have to address the link between N1 and estimation in detail.

### Mid-Latency Neural Activities and Subitizing

Recent studies (e.g., Drew and Vogel, 2008; Mazza and Caramazza, 2011; Ester et al., 2012; Anderson et al., 2013a) have focused on the relationship between object numerosity and a mid-latency lateralized posterior component of the EEG activity—the N2pc—which typically emerges at approximately 200 ms. The N2pc represents a difference in activation between the posterior areas contralateral to the target side relative to the ipsilateral areas. This component has traditionally been measured during visual search tasks, when a single target is presented among distracters (e.g., Luck and Hillyard, 1994; Eimer, 1996; Jolicoeur et al., 2006; Töllner et al., 2012; Eimer and Grubert, 2014), and was originally interpreted as indexing distracter suppression (Luck et al., 1997). Other studies (Eimer, 1996; Hickey et al., 2009) have challenged the notion that the stage of processing reflected in this neural activity indicates distracter suppression, and some (Mazza et al., 2009) have proposed that it may reflect a feature-to-location binding mechanism that results in enhanced individuation of the relevant (target) object.

Studies on enumeration (e.g., Mazza and Caramazza, 2011, 2012; Ester et al., 2012) have additionally shown that N2pc is modulated specifically by the numerosity of the targets presented in visual displays where the overall item number was kept constant, highlighting the link between attention selection and multiple object individuation. The asymptote of the modulation of this response at approximately 3–4 elements (Ester et al., 2012; Pagano and Mazza, 2012) indicates a capacity limit in simultaneous object individuation that mirrors the behavioral subitizing effect. Furthermore, the neural asymptote correlates with individual subitizing limits as seen from behavioral performance (Ester et al., 2012; Pagano et al., 2014). Importantly, the modulation of the N2pc as a function of target numerosity is independent of the presence of distracters (Mazza et al., 2013), thus further highlighting the link between the representation generated at this stage of processing and exact enumeration (Figure 2). Finally, the fact that N2pc is modulated by low-level factors, such as target grouping by color (Mazza and Caramazza, 2012), but not by the numerical identity of target elements (e.g., using digits as elements to be counted, such as presenting two “3s” does not affect the N2pc; Pagano and Mazza, 2013) further characterizes individuation as a perceptual pre-numeric stage that operates separately from brain structures involved in processing the semantic numerical value of the target set.

The numerosity-related modulation of N2pc has also been found in other tasks requiring simultaneous processing of multiple objects, such as tracking of multiple moving objects (Drew and Vogel, 2008) and working memory tasks (Anderson et al., 2013a,b). For instance, Drew and Vogel (2008) found numerosity-related N2pc modulation and asymptote when a varying number of static targets had to be selected before they started moving. These results suggest that the attention-based mechanism of object individuation is a core component of the visual system involved in processing multiple targets in a variety of tasks, and not only enumeration. These results resonate with recent fMRI findings (Knops et al., 2014), which indicate that the same areas in the parietal cortex are active during the execution of enumeration and working memory tasks, thus suggesting that different tasks involving multiple objects are subserved by a single mechanism. However, the N2pc modulation is not merely triggered by the presence of multiple targets. Indeed, when the task is simply to report the presence of at least one target, namely in a detection task where there is no specific need to keep the relevant elements distinguished from each other, an N2pc is still elicited but with no numerosity-related modulation (Mazza and Caramazza, 2011). This characterizes object individuation as a flexible mechanism whose functioning is adjusted to meet specific task demands.

Overall, we interpret this set of results as evidence for an attention-based object individuation mechanism that makes available a representation of either a single or a limited set of objects. This representation forms the crucial input for further cognitive operations and is the appropriate one for exact enumeration. These findings also converge with recent behavioral studies associating subitizing with attention (Vetter et al., 2008; Burr et al., 2010), and with neuroimaging studies (Ansari et al., 2007; Vetter et al., 2011; Cutini et al., 2014) implicating the involvement of areas typically related to stimulus-driven attention (e.g., the tempo-parietal junction) in the subitizing phenomenon and in exact enumeration in general. Future research will address the contribution of stimulus-driven vs. goal-directed aspects, as well as their interaction, in the modulation of this mid-latency EEG response.

### Late Neural Modulation of Object Numerosity

A later sustained EEG response occurring at approximately 300 ms—Contralateral Delayed Activity, CDA; often referred to as Sustained Posterior Negativity- is also modulated by target quantity (e.g., Vogel and Machizawa, 2004; Jolicoeur et al., 2006; Drew and Vogel, 2008; Anderson et al., 2011). This response has typically been measured in tasks in which participants have to memorize a varying number of elements for a subsequent match-to-sample test, and for this reason it has been interpreted as the index of the active maintenance of a limited set of elements in a working memory buffer (Vogel and Machizawa, 2004). The fact that the CDA amplitude also asymptotes at around 3–4 elements suggests the possibility that the limits in accurate enumeration may reflect the capacity limits of working memory. This hypothesis was discussed in the seminal studies on the subitizing phenomenon (see Trick and Pylyshyn, 1993 for a discussion), and has been reconsidered by more recent studies (Tuholski et al., 2001; Piazza et al., 2011; Cutini and Bonato, 2012).

EEG studies have confirmed that the numerosity-related modulation and asymptote in the CDA component also extend to enumeration tasks (Mazza and Caramazza, 2011; Pagano et al., 2014; Figure 2). This result indicates the involvement of working memory in exact enumeration, reflecting the active maintenance of the selected objects in visual working memory for mapping onto a symbolic numerical value. Nonetheless, the temporal sequence of the occurrence of this EEG response indicates that working memory capacity limits is not the ultimate cause of the subitizing phenomenon—a capacity limit is already apparent at the earlier N2pc stage. Thus, we propose that the neural modulation at this late stage of stimulus processing represents a byproduct of the functioning of the individuation mechanism.

## Conclusions and Future Directions

We have proposed that recent EEG research on multiple object processing, along with recent behavioral (Railo et al., 2008; Vetter et al., 2008; Burr et al., 2010) and neuroimaging studies (Ansari et al., 2007; Xu and Chun, 2009; Vetter et al., 2011; Knops et al., 2014), supports the view that the core component of exact enumeration is an attention-based individuation mechanism that binds specific features to locations and provides a stable representation of a limited set of relevant objects. The lateralized nature of the mid-latency N2pc component (a proxy for location coding),^1^ its asymptote at about 3 targets (an index of limit in simultaneous processing), and the fact that it is found both when distracters are and when they are not present (indicating selective individuation of the target elements) support this interpretation. Additionally, the result of an N2pc modulation in the absence of distracters reasonably dismisses the possibility that this pattern is exclusively related to distracter suppression.

The existing results on enumeration and multiple object tracking suggest that the attention-based mechanism of object individuation indexed by the N2pc is shared by different tasks involving multiple target processing. However, it is currently unclear what are the core properties that allow for individuation, and if these are always the same across various tasks. In particular, what exactly is individuated during enumeration? Influential theories of vision (Pylyshyn, 2001) propose that individuation operates over “separate” entities. However, it is unknown whether this implies that the entities be spatially isolated from one another, as is the case of separate objects, or whether individuation, and the resulting subitizing effect, can occur over separate features of a single object (Porter et al., 2013).

In this review we have discussed evidence supporting the idea that the individuation mechanism is separate from the one that is reflected in the early N1 component. On the basis of the N1 pattern found in previous studies (Nan et al., 2006; Libertus et al., 2007; Hyde and Spelke, 2009; Mazza et al., 2013), indicating a modulation related to the overall increase in the number of elements in the visual field, we argue that the representation generated at this level is based on the “spatial” separability of the objects, but without a precise feature coding of the elements. For this reason, we speculate that this stage of processing may provide the basic information sufficient for approximate enumeration (but see Hyde, 2011).^2^

Here we have interpreted the observed modulation of N1 to reflect sensitivity to object numerosity. However, there is a potential confound related to the fact that variation in object numerosity covaries with variation in visual parameters such as, for example, the individual dot size, total area or density of the stimulus configuration (for an example of the effect of density on the N1 see Gebuis and Reynvoet, 2012; for a discussion see Gebuis et al., 2014; Soltész and Szucs, 2014 but see Hyde and Spelke, 2009, where individual dot size and interim spacing were equated on the critical test trials). Future work will have to address the possibility that the N1 modulation reflects sensitivity to variation in the visual parameters of the stimulus configuration, rather than tracking object numerosity *per se*. Independently of how this issue is resolved, it is important to note that changes in total area cannot provide a good account for the pattern of results found for the N2pc, given that its numerosity-related modulation is found also when the overall number of elements (and therefore, the total area) is kept constant (see Mazza et al., 2013).

Our review also indicates the involvement of a working-memory component in the subitizing phenomenon, as reflected in the CDA modulation. However, the time-wise information provided by EEG measurements allows us to establish the temporal sequence of the occurrence of the individuation and working memory stages, and to point out the key role of individuation for subitizing. We propose that the representation generated by the neural structures underlying the N2pc serves as input for a working memory mechanism, reflected in the CDA, which enables the active maintenance of the individuated set of items while mapping the elements onto a symbolic quantity value. As such, it is the limit in the simultaneous individuation of objects that ultimately determines the subitizing limit.

Thus far we have considered the subitizing effect as having a fixed limit at approximately 3–4 items, with the only variation due to individual differences. In contrast, recent studies (Haladjian and Pylyshyn, 2011; Haladjian and Mathy, 2015) have found that the subitizing limit is modulated by task report, with the larger numerosity limit in localization tasks compared to enumeration interpreted as reflecting the global spatial apprehension of the stimulus configuration. This interpretation of the observed modulation of the subitizing limit seems to suggest a link between this effect and the mechanism reflected in the N1 response, but confirmation will have to await future research.

In conclusion, we have proposed a framework for the interpretation of the various stages involved in multiple object processing during exact enumeration (Figure 2). In particular, we have isolated three separate mechanisms that track the numerosity of the elements presented in the visual field for different purposes. An early mechanism (100 ms post-stimulus onset, reflected in the N1) operates over the entire configuration, and allows for volatile representations of the number of elements, irrespective of their relevance for the task at hand. A mid-latency mechanism (200 ms post-stimulus, reflected in the N2pc) distinguishes the relevant elements from surrounding distracters, and simultaneously individuates up to 3–4 relevant elements. The resulting enriched representation of the set of elements subsequently feeds a working memory buffer (CDA, 300 ms post-stimulus), in which the individuated objects are actively retained during quantity-to-symbol mapping.

This framework offers the basis for new research approaches on multiple object processing. For example, it could be used to plan studies directed at characterizing enumeration and multiple object processing throughout lifespan. While this aspect has been investigated intensively in infants and children (e.g., Leslie et al., 1998; Feigenson et al., 2004), it is currently unclear whether and how enumeration abilities undergo changes in aging. Since the ability to process multiple objects simultaneously is essential to perform many tasks in everyday life, evaluating how it is modified by aging is important to assess the integrity of the cognitive functions in older individuals. Our framework would help to individuate the age-related changes in the specific subcomponents that are sensitive to object numerosity.

## Conflict of Interest Statement

The authors declare that the research was conducted in the absence of any commercial or financial relationships that could be construed as a potential conflict of interest.

## Footnotes

^1^While this aspect of the paradigm helps establish a location-based process, it also reflects a potential limitation in the generality of the phenomenon in that it is associated with a paradigm that requires the presentation of lateralized targets.

^2^The point here is not that representations formed at the N1 stage could not be used for exact enumeration. This would only be possible for the special case where the “targets” are either the only items or they are spatially segregated in the visual field, namely when the elements to be enumerated are not spatially intermingled with other objects. However, the subitizing effect is visible also when targets are presented in cluttered scenes (see Trick and Pylyshyn, 1993; Watson et al., 2007).
